# Hypoxia Promotes Glycogen Accumulation through Hypoxia Inducible Factor (HIF)-Mediated Induction of Glycogen Synthase 1

**DOI:** 10.1371/journal.pone.0009644

**Published:** 2010-03-12

**Authors:** Nuria Pescador, Diego Villar, Daniel Cifuentes, Mar Garcia-Rocha, Amaya Ortiz-Barahona, Silvia Vazquez, Angel Ordoñez, Yolanda Cuevas, David Saez-Morales, Maria Laura Garcia-Bermejo, Manuel O. Landazuri, Joan Guinovart, Luis del Peso

**Affiliations:** 1 Departamento de Bioquímica, Universidad Autónoma de Madrid, Madrid, Spain; 2 Instituto de Investigaciones Biomédicas “Alberto Sols”, Consejo Superior de Investigaciones Científicas, Madrid, Spain; 3 Institute for Research in Biomedicine (IRB Barcelona), University of Barcelona, Barcelona, Spain; 4 Department of Biochemistry and Molecular Biology, University of Barcelona, Barcelona, Spain; 5 Servicio de Inmunología Hospital de la Princesa, Universidad Autónoma de Madrid, Madrid, Spain; 6 Servicio de Anatomía Patológica Hospital Ramón y Cajal, Madrid, Spain; Institute of Genetics and Molecular and Cellular Biology, France

## Abstract

When oxygen becomes limiting, cells reduce mitochondrial respiration and increase ATP production through anaerobic fermentation of glucose. The Hypoxia Inducible Factors (HIFs) play a key role in this metabolic shift by regulating the transcription of key enzymes of glucose metabolism. Here we show that oxygen regulates the expression of the muscle glycogen synthase (GYS1). Hypoxic GYS1 induction requires HIF activity and a Hypoxia Response Element within its promoter. GYS1 gene induction correlated with a significant increase in glycogen synthase activity and glycogen accumulation in cells exposed to hypoxia. Significantly, knockdown of either HIF1α or GYS1 attenuated hypoxia-induced glycogen accumulation, while GYS1 overexpression was sufficient to mimic this effect. Altogether, these results indicate that GYS1 regulation by HIF plays a central role in the hypoxic accumulation of glycogen. Importantly, we found that hypoxia also upregulates the expression of UTP:glucose-1-phosphate urydylyltransferase (UGP2) and 1,4-α glucan branching enzyme (GBE1), two enzymes involved in the biosynthesis of glycogen. Therefore, hypoxia regulates almost all the enzymes involved in glycogen metabolism in a coordinated fashion, leading to its accumulation. Finally, we demonstrated that abrogation of glycogen synthesis, by knock-down of GYS1 expression, impairs hypoxic preconditioning, suggesting a physiological role for the glycogen accumulated during chronic hypoxia. In summary, our results uncover a novel effect of hypoxia on glucose metabolism, further supporting the central importance of metabolic reprogramming in the cellular adaptation to hypoxia.

## Introduction

Aerobic glycolysis yields sixteen times more ATP than glucose fermentation. Thus, metazoans are heavily dependent on the highly energy-efficient aerobic metabolism to meet their large ATP demands. Since oxygen is the final electron acceptor during mitochondrial respiration, this element is essential for metazoan metabolism. In fact, most of the oxygen consumed by animals is required to sustain oxidative phosphorylation. However, several animal species are able to adapt to reduced oxygen tensions during variable periods of time [1]. These include diving animals, fossorial mammals, animals living at high altitude and species of fish that live in stagnant waters, among others [1]. In nearly all these cases, the adaptation to hypoxia involves systemic and cellular responses aimed to reduce oxygen consumption and optimize its utilization.

At the cellular level, the reduction in oxygen consumption is mediated by a shift from oxidative to fermentative glucose metabolism [2], [3], [4], [5], the fine-tuning of mitochondrial respiration [6] and an adjustment of mitochondria number. This hypometabolic state is achieved by the induction of a specific gene expression program under the control of the Hypoxia-Inducible Factor (HIF) family of transcription factors. This family comprises three transcription factors (HIF-1, HIF-2 and HIF-3) that are heterodimers of a constitutively expressed β subunit and an oxygen-sensitive α subunit (HIF1α, HIF2α or HIF3α) [7], [8], [9]. In the presence of oxygen, HIFα is extremely unstable due to an oxygen-dependent posttranslational hydroxylation that targets it for proteosomal degradation [10], [11], [12], [13], [14]. A family of prolyl hydroxilases (EGLNs), that require molecular oxygen as a reaction cosubstrate, catalyzes the hydroxylation of HIFα [15], [16], [17]. Hence, under hypoxia, hydroxylation is compromised leading to HIF stabilization and, subsequently, to its binding the hypoxia-response element (RCGTG motif) on the regulatory region of target genes [18]. Among these genes are those of glucose transporter SLC2A1 (Glut1) and almost all glycolytic enzymes [2], whose induction leads to an increased glycolytic flux. In addition, hypoxia regulates the end product of glycolysis, pyruvate, which is actively diverted from mitochondrial oxidation due to the HIF-mediated induction of Pyruvate Dehydrogenase Kinases (PDK1 and PDK4) [3], [4], [19]. Finally, HIF also induces the transcription of lactate dehydrogenase (LDH) [20] and lactate transporter (MCT4) [21], whose activities account for the transformation of pyruvate into lactate and its export out of the cell, respectively. Overall, the coordinated induction of all these genes by HIF decreases glucose oxidation, contributing to reduced oxygen consumption, and increases anaerobic ATP production to maintain energy balance. Thus, the adaptation to hypoxia is critically dependent on the reprogramming of glucose metabolism and, accordingly, a large number of HIF-target genes function in coordination to promote this metabolic shift. Importantly, because of all these metabolic adaptations, hypoxic cells rely heavily on an appropriate glucose supply. To ensure glucose availability, cells that depend on anaerobic glycolysis to sustain ATP production, such as skeletal myotubes, accumulate large amounts of glucose in the form of glycogen. In agreement, some reports described an increase in glycogen upon exposure of cells to hypoxia [22], [23] or hypoxia-mimetics [24]. However, in spite of these pieces of evidence, the effect of hypoxia on glycogen metabolism and the molecular mechanism involved are yet to be addressed.

Herein we demonstrate that hypoxia leads to glycogen accumulation through the coordinated regulation of glycogen biosynthesis. We show that GYS1 is a novel hypoxia-inducible, HIF-dependent gene, whose induction mediates glycogen accumulation following oxygen deprivation. Consequently, this work reveals a novel metabolic response to low oxygen and emphasizes the relevance of glucose metabolism in the adaptation to hypoxia. Furthermore, we found that GYS1 interference abrogates hypoxic preconditioning in an *in vitro* model of ischemia. We hence propose that the HIF-dependent increase in glycogen stores is a hypoxia-triggered adaptation that prepares cells to cope with further oxygen restrictions by ensuring adequate substrate supply for anaerobic glycolysis.

## Results

### Hypoxia induces GYS1 expression in a HIF-dependent manner

**Figure 1:**
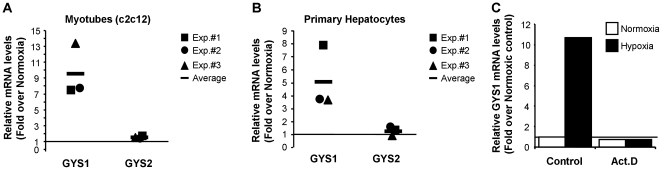
GYS1, but not GYS2, is a hypoxia-regulated HIF target gene. c2c12 myotubes (A) or primary mouse hepatocytes (B) were exposed to normoxia or hypoxia for 12 hours and the level of GYS1 and GYS2 mRNA was determined by quantitative PCR. The amount of each mRNA in samples was normalized to the content of β-actin mRNA in the same sample. The graph represents the fold values of hypoxic over normoxic mRNA levels normalized to the value of 1 (horizontal line). Data shown are the results of three independent experiments and their mean. (C) c2c12 cells were treated with 1 µg/ml actinomycin D (Act.D) or vehicle (control) for 30 minutes and then exposed to normoxia or 1% oxygen (Hypoxia) for 12 hours. The graph represents GYS1 mRNA levels in each condition relative to the untreated (control) normoxic cells. The experiment was repeated twice with identical results.

**Figure 2:**
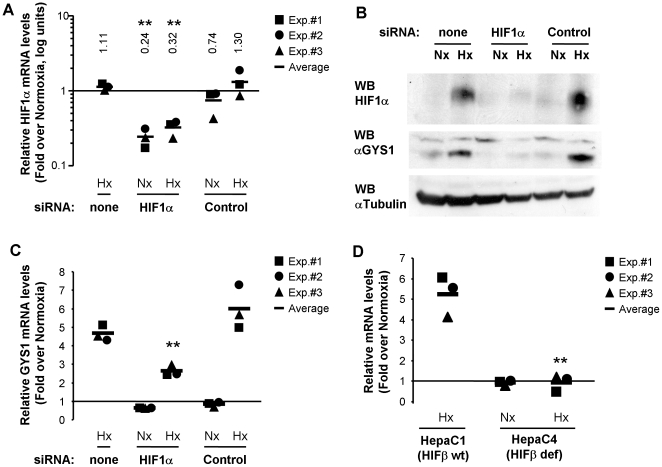
HIF mediates GYS1 induction by hypoxia. c2c12 myoblasts were transfected with siRNA against mouse HIF1α (HIF1α), an siRNA containing an irrelevant sequence (Scramble) or left untreated (none). 24 hours after transfection cells were cultured for 12 hours at 21% (Nx) or 1% oxygen (Hx) and then processed to determine mRNA (A,C), or protein (B) levels. Graphs represent normalized mRNA levels in each condition relative to the untreated normoxic cells. Data shown are the results of three independent experiments and their mean. **, p<0.01 as compared to samples transfected with irrelevant siRNA (Control) and exposed to the same oxygen tension. Numbers represent average values. (D) HIF-competent (HIFβ wt), Hepa C1, and HIF-deficient (HIFβ def), Hepa C4, cells were exposed to normoxia (Nx) or 1% oxygen (Hx) for 12 hours and processed to determine GYS1 mRNA level. The graph represents the normalized GYS1 mRNA levels in each condition relative to the normoxic Hepa C1 cells (horizontal line) from three independent experiments. **, p<0.01 as compared to HIF-competent cells exposed to the same oxygen tension.

With the aim of understanding the whole array of metabolic adaptations that cells employ to cope with reduced oxygen tension, we searched the human genome for the occurrence of potential hypoxia-response elements (HREs). This search was based on the evolutionary conservation of HREs and their similitude to known functional HRE-containing regulatory regions [25]. The details of the strategy were reported elsewhere (Ortiz et al. Nucl. Acids Res. *in press*). One of the potential HREs identified by this approach was located in the promoter of the muscle isoform of glycogen synthase, GYS1. To investigate the potential regulation of GYS1 by hypoxia, we exposed c2c12 skeletal muscle myotubes to 1% oxygen and determined the level of GYS1 mRNA by quantitative PCR. Figure 1A shows that GYS1 mRNA was strongly induced by hypoxia in c2c12 cells. In contrast, hypoxia did not alter the mRNA levels of the closely related GYS2 (liver glycogen synthase) enzyme (figure 1A). In agreement, we did not find evolutionarily conserved HREs in the GYS2 locus. The hypoxic induction of GYS1 was not restricted to differentiated skeletal muscle cells, but was also observed in myoblast as well as other primary and stable cell lines (Figure S1 and data not shown). It is noteworthy that hypoxia also led to GYS1 induction in the hepatoma cell lines HepG2 (data not shown) and HepaC1 (see below), as well as in primary hepatocytes (figure 1B). The induction of GYS1 mRNA was inhibited by actinomycin D treatment (figure 1C), suggesting that hypoxia affected its transcription rather than its stability. Altogether, these results indicated that GYS1 was a hypoxia-inducible gene, and strongly hinted that it was also a HIF-target gene. In agreement, treatment of c2c12 cells with a siRNA targeting HIF1α (figure 2A and 2B) reduced GYS1 induction by hypoxia (figure 2B and C). The partial induction of GYS1 by hypoxia observed in HIF1α knock down cells could be explained by the remaining HIF1α expression and/or the contribution of HIF2α to GYS1 regulation. To corroborate the implication of HIF in this process, we investigated the effect of hypoxia on GYS1 expression in the HepaC4 HIF-deficient cells (figure 2D). These cells contain a mutant HIFβ subunit with a severely reduced half life and, as a consequence, they are deficient in HIF activity [26], [27]. As shown in figure 2D, while hypoxia induced glycogen synthase accumulation in the parental HIF-competent HepaC1 cells, this effect was completely blunted in the HepaC4 cells (figure 2D). Taken together, these results identify GYS1 as a novel HIF-target gene upregulated by hypoxia.

### GYS1 promoter region contains a functional HRE

**Figure 3:**
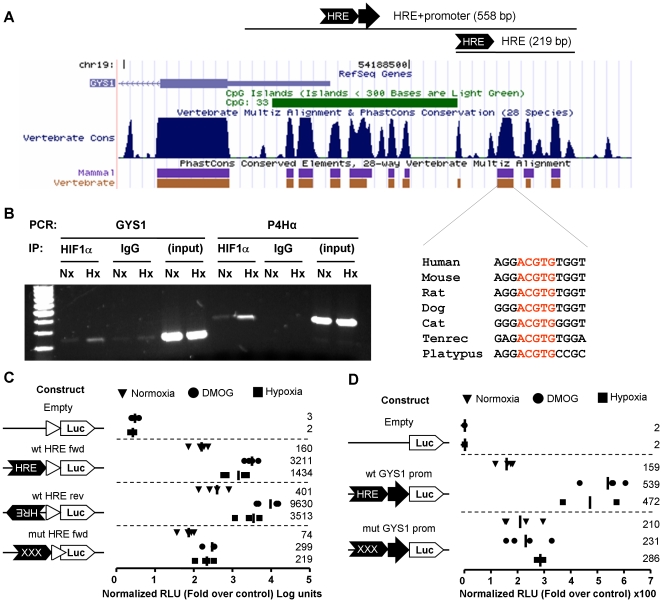
Identification of a functional HRE in GYS1 genomic region. (A) Schematic representation of human GYS1 genomic region from -429 to +298 showing evolutionarily conserved regions. Adapted from the UCSC Genome Browser (http://genome.ucsc.edu/). The lines above the graph show the genomic regions cloned in the indicated reporter constructs (see text). The inset under the figure represents the sequence alignment of the HRE element from the indicated species. (B) c2c12 cells exposed to 21% (Nx) or 1% (Hx) oxygen for 12 hours and then fixed and processed for chromatin immunoprecipitation with anti-HIF1α (HIF1α) antibodies or control IgG (IgG). (Input) lanes correspond to total sonicated genomic DNA. After immunoprecipitation, chromatin was amplified with primers specific for the HRE-containing genomic regions from GYS1. The HIF binding region within the Procollagen 4-hydroxylase α (P4Hα) was included as a positive control [25]. (C, D) HeLa cells transfected with a reporter plasmid containing GYS1 genomic region (-210 to -419) upstream of a minimal promoter (C) or a plasmid containing the whole GYS promoter region (+128 to -419) driving the expression of a luciferase reporter gene (D). Where indicated (mutHRE) the consensus HRE sequence (ACGT) was mutated to TAGC. The graphs represent the corrected luciferase activity values of each construct over the luciferase activity obtained in normoxic cells transfected with empty plasmids (control). Data shown are the results of four independent experiments and their mean (bar and numbers on the right).

The HRE identified by our bioinformatic search (Ortiz et al. Nucl. Acids Res. *in press*) was located at position -314 in the promoter region of human GYS1 (figure 3A). This site matched the canonical ACGTG motif and was conserved across mammals (figure 3A). Moreover, the HRE motif was included in a non-coding region conserved across vertebrates (figure 3A, PhastCons vertebrate track) [28]. Importantly, HIF1α associated with the genomic region containing this HRE motif upon exposure of c2c12 cells to hypoxia (Figure 3B), suggesting that, in intact cells, HIF binds this site. To test the functionality of this HRE, we cloned the region from -211 to -429 upstream a minimal promoter driving the expression of the firefly luciferase reporter gene. As shown in figure 3C, hypoxia and the EGLN inhibitor DMOG strongly induced luciferase activity in cells transfected with the construct containing the potential HRE regardless of its orientation. Importantly, mutation of the HRE motif completely impaired the induction of this construct by hypoxia (figure 3C). Next, we tested whether this element was able to confer hypoxia-inducibility to the native GYS1 promoter. To this end, we cloned the whole promoter region of the human GYS1 gene including the HRE (+128 to –429) into the pGL3-basic plasmid. This genomic region showed promoter activity since it drove luciferase expression above pGL3 basal levels (figure 3D). Significantly, the promoter activity of this construct was robustly induced by hypoxia and DMOG (figure 3D). In addition, the induction by Hypoxia and DMOG, but not the basal promoter activity, was lost when the reporter construct included a mutation in the HRE motif (figure 3D). In summary, GYS1 contains an evolutionarily conserved HRE located at -314 that mediates its induction by hypoxia.

### Hypoxia-mediated induction of GYS1 results in increased glycogen synthase activity

**Figure 4:**
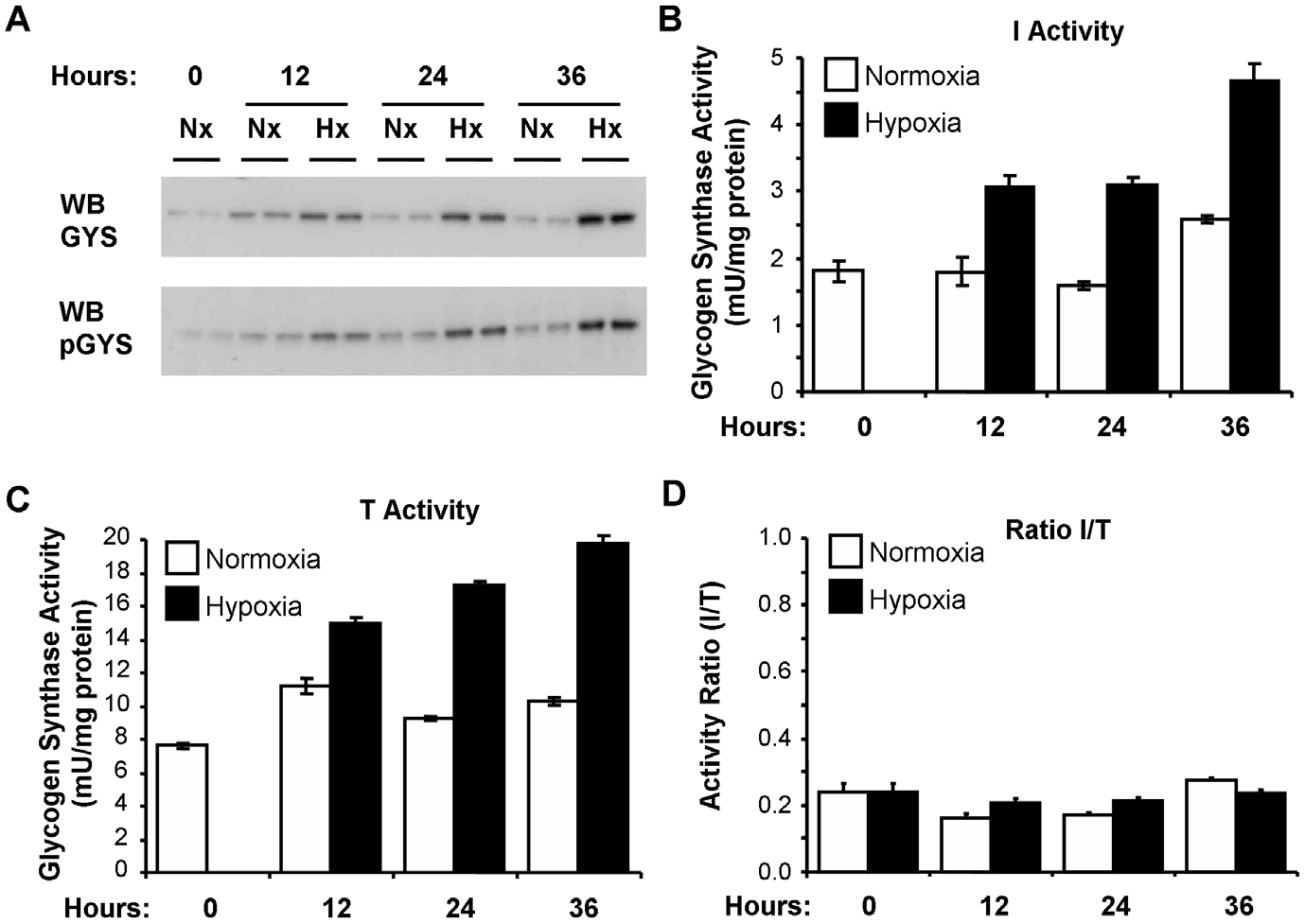
Hypoxia increases glycogen synthase activity. c2c12 myoblasts were incubated at 21% (Nx) or 1% oxygen (Hx) for the indicated periods of time and processed to determine GYS1/pGYS1(phospho-Ser 641) levels (A) and glycogen synthase activity in the absence (B) or presence of 6.6 mM of the allosteric modulator glucose-6-phospate (C). Graphs represent the mean of duplicated measures and error bars the range. (D) The graph represents the ratio of glycogen synthase activity in the absence (I) and presence (T) of glucose-6-phosphate. The experiment was repeated twice with similar results.

The experiments described above demonstrated that hypoxia induced GYS1 transcription in a HIF-dependent manner. Importantly, gene transcription correlates with an increment of muscle glycogen synthase protein (figure 2B and 4A). Since glycogen synthase (from now on GS) activity is subjected to a complex regulation by allosteric modulators and phosphorylation by several kinases, protein levels do not necessarily correlate with activity. Therefore, we next asked whether the increment in GYS1 protein resulted in augmented GS activity. Figure 4B shows that GS activity increased in parallel with protein accumulation (figure 4A). Importantly, in the presence of the allosteric activator glucose-6-phosphate, that drives conversion of GS to its fully active state, samples from hypoxic cells still displayed increased GS activity as compared to control samples (figure 4C). Thus, hypoxia increased the total amount of GS but did not alter the proportion of active/inactive conformations as shown by the I/T ratio (figure 4D). In agreement, hypoxia did not modify the fraction of phosphorylated GYS1 (figure 4A). These results strongly suggest that GYS1 induction by HIF leads to augmented GS activity.

### Hypoxia induces glycogen accumulation

**Figure 5:**
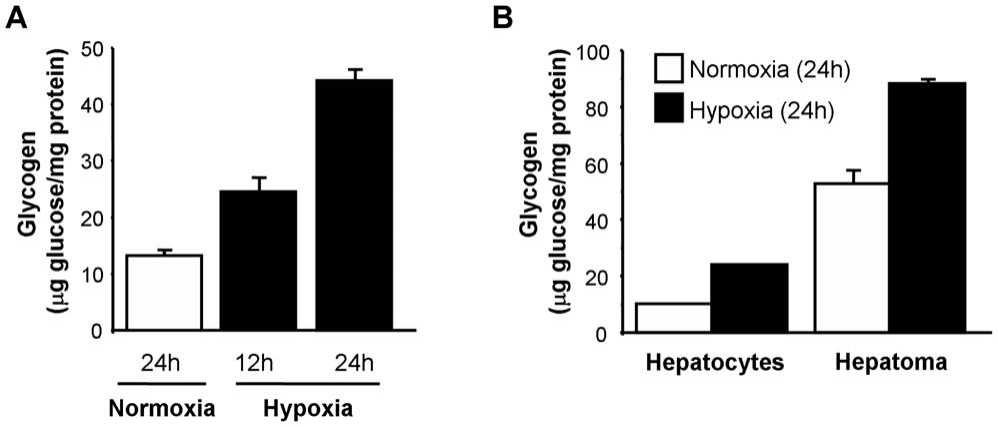
Hypoxia induces glycogen accumulation. C2c12 myotubes (A), inmortalized mouse hepatocytes (B) and mouse hepatoma Hepa C1 cells (B), were exposed to normoxia or hypoxia (1% Oxygen) for the indicated periods of time and glycogen content was determined. Graphs represent average measures of glycogen content in two independent samples and error bars the range. Data shown are representative of at least five (A) or two (B) independent experiments.

In order to test the functional relevance of increased GS activity, we exposed c2c12 myotubes to hypoxia (1% oxygen) or normoxia (21% oxygen) and determined their glycogen content at different time points. As shown in figure 5A, hypoxia resulted in a significant increase in glycogen concentration. Significantly, as noted for GYS1 gene induction (figure 1), glycogen accumulation was not restricted to muscle cells as it was also observed in liver-derived cells (figure 5B). Collectively, these results indicate that the hypoxic induction of GYS1 correlates with a significant increase in glycogen reserves.

### Hypoxia-induced glycogen accumulation is HIF-dependent

**Figure 6:**
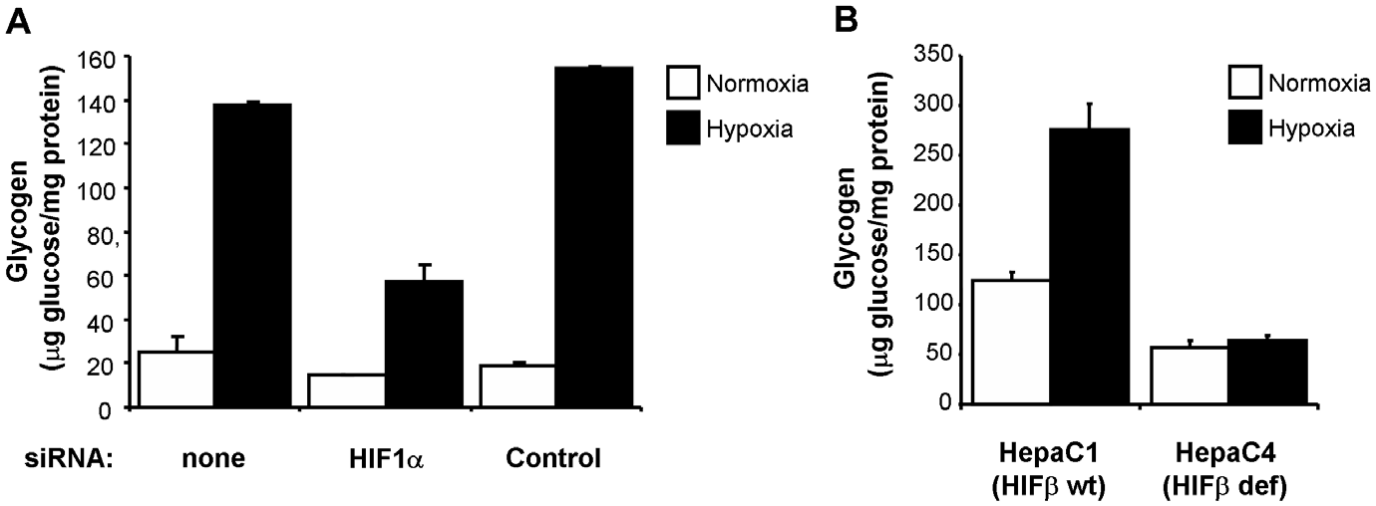
Hypoxia-driven glycogen accumulation is HIF-dependent. (A) c2c12 myoblasts were transfected with siRNA against mouse HIF1α (HIF1α), siRNA containing an irrelevant sequence (Control) or left untreated (none). After transfection cells were cultured for 12 hours under normoxia or 1% oxygen (Hypoxia) and processed to determine glycogen content. Graphs represent the average of two independent determinations and their range. Data shown are representative of three independent experiments. (B) Hepa C1 and Hepa C4 cells were exposed to 21% (Normoxia) or 1% oxygen (Hypoxia) for 12 hours and processed to determine their glycogen content. Graphs represent the mean of duplicated measures and error bars the range, results shown are representative of three independent experiments.

As discussed before, HIF mediates most of the metabolic adaptations to hypoxia. Therefore, we next investigated the role of this transcription factor in glycogen accumulation. As shown in figure 6A, reduction of HIF activity by siRNA treatment attenuated the hypoxia-induced glycogen accumulation. In addition, the use of HepaC1/C4 cells provided further evidence of the role of HIF on the hypoxic accumulation of glycogen. While hypoxia induced glycogen accumulation in the HIF-competent HepaC1 cells, this effect was abrogated in the HIF-deficient HepaC4 cells (figure 6B). These results demonstrate that HIF is necessary for the accumulation of glycogen induced by hypoxia.

### GYS1 mediates the hypoxic accumulation of glycogen

**Figure 7:**
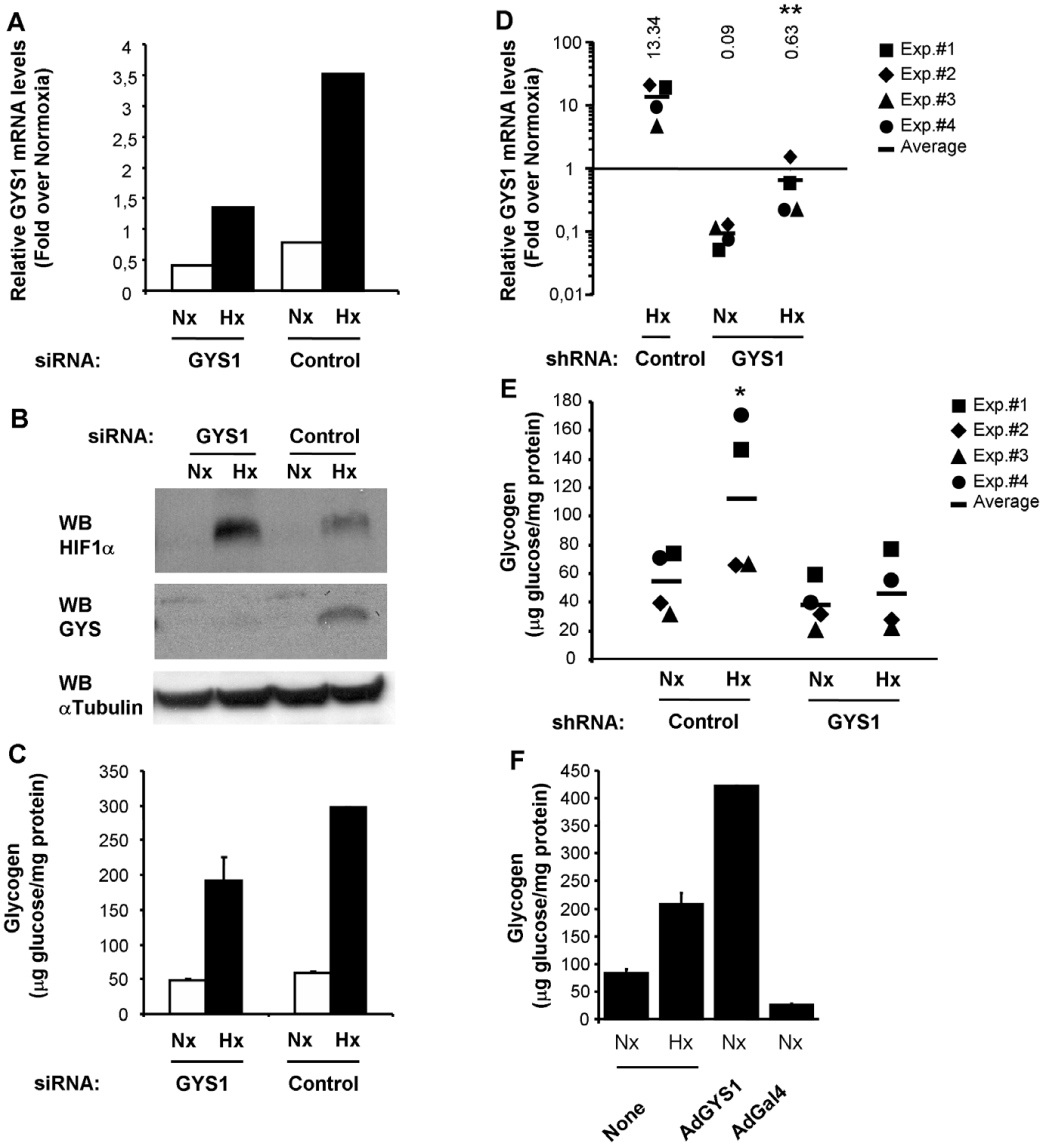
GYS1 mediates glycogen accumulation during hypoxia. c2c12 myoblasts were transfected with siRNA against mouse GYS1 (GYS1), siRNA containing an irrelevant sequence (Control). 24 hours after transfections cells were cultured for 12 hours under 21% (Nx) or 1% oxygen (Hx), then each sample was split and processed to determine GYS1 mRNA (A), protein levels (B) or glycogen content (C). The experiment was repeated twice with similar results. GYS1 mRNA levels (D) and glycogen content (E) were determined in Hepa C1 cells stably expressing a shRNA directed to GYS1 or a control shRNA. Results from four independent experiments are represented. *, p<0.05; **, p<0.01; as compared to samples expressing control shRNA and exposed to normoxia. (F) c2c12 myotubes were infected with AdGYS1 or Adβ-Gal. 24 hours after infection cells were processed for glycogen content determination. Parallel cultures were exposed to normoxia or hypoxia for 12 hours and processed for glycogen determination. Graphs represent the mean of duplicated measures and error bars the range, the experiment was repeated twice with similar results.

The results presented above demonstrate that hypoxia results in an increase in GYS1 expression and total glycogen synthase activity that correlates with glycogen accumulation. In order to establish a functional link between these phenomena, we investigated the effect of GYS1 interference on hypoxia-induced glycogen accumulation. Treatment of c2c12 with siRNA against GYS1 resulted in a reduction of both GYS1 mRNA and protein (figures 7A and 7B) that negatively affected glycogen accumulation induced by hypoxia as compared to control cells transfected with scramble siRNA (figure 7C). Since GS activity is an absolute requirement for glycogen synthesis, we next investigated the effect of knocking down GYS1 in the hepatoma HepaC1 cells, which also express the liver isoform GYS2. To this end, we infected HepaC1 cells with a retrovirus encoding for a short hairpin RNA directed to GYS1 (shGYS1) or an irrelevant shRNA (control), and selected pools of cells that stably integrated the construct. Expression of shGYS1 reduced GYS1 mRNA expression to 12±7.7% of control cells expressing a control shRNA (figure 7D) without affecting GYS2 expression (GYS2 mRNA level was 67±16% of that in control cells). In agreement with the experiments in c2c12 cells, knockdown of GYS1 prevented the hypoxia-induced accumulation of glycogen (figure 7E). Finally, we asked whether GYS1 induction was sufficient to promote glycogen accumulation. To this end, we determined the glycogen content of c2c12 cells infected with an adenovirus encoding for GYS1. As shown in figure 7F, forced expression of GYS1 led to the accumulation of glycogen to levels similar as those obtained under hypoxia. Altogether, these experiments indicate that GYS1 induction by HIF could explain the accumulation of glycogen observed under hypoxia.

### Hypoxia regulates glycogen metabolism at multiple levels to promote glycogen accumulation

**Figure 8:**
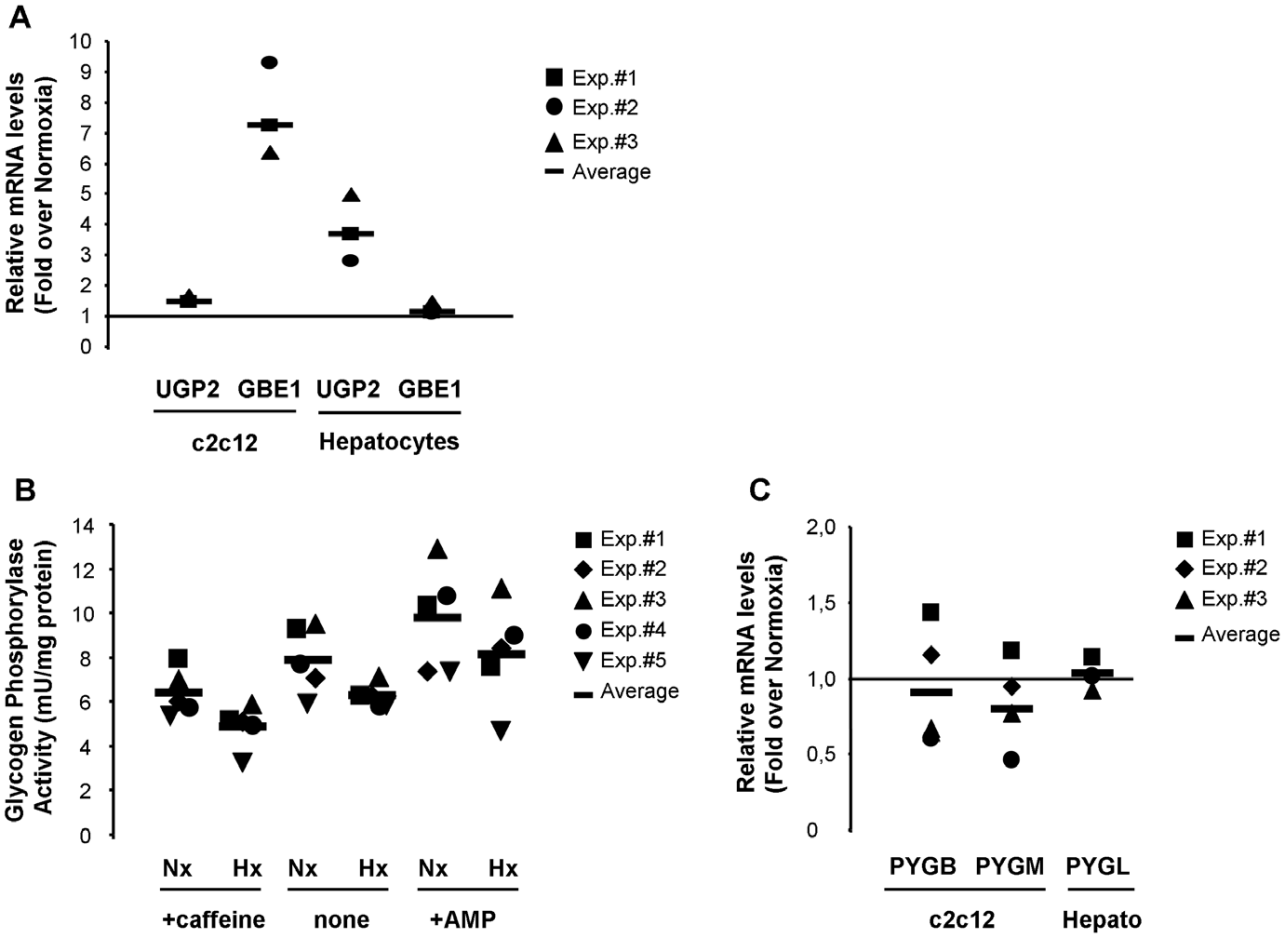
Hypoxia affects glycogen metabolism at multiple levels. (A) c2c12 myoblast and primary mouse hepatocytes were exposed to normoxia or hypoxia for 12 hours and the level of UGP2 and GBE1 mRNA was determined by quantitative PCR. The amount of each mRNA in samples was normalized to the content of β-actin mRNA in the same sample. Data shown represent the fold values of hypoxic over normoxic mRNA levels normalized to the value of 1 (horizontal line) in three independent experiments. (B) c2c12 myoblasts were exposed to normoxia (Nx) or hypoxia (Hx) for 24 hours and the activity of glycogen phosphorylase was assayed in untreated cell lysates, or in the presence of cAMP or caffeine. The results from five independent experiments and their mean are shown. (C) c2c12 myoblasts and primary mouse hepatocytes (Hepato) were exposed to normoxia or hypoxia for 12 hours and the level of PYGM, PYGB and PYGL mRNA was determined by quantitative PCR. The amount of each mRNA in samples was normalized to the content of β-actin mRNA in the same sample. Data shown represent the fold values of hypoxic over normoxic mRNA levels normalized to the value of 1 (horizontal line) in three independent experiments.

The effect of hypoxia on glucose catabolism is mediated through the induction of almost all the enzymes of the glycolytic pathway in a coordinated fashion. Thus, we next investigated whether hypoxia modulated other enzymes involved in glycogen synthesis. In fact, in addition to GYS1, our bioinformatics strategy (Ortiz et al. Nucl. Acids Res. in press) also identified UGP2 and GBE1 as potential HIF targets. Specifically we found two potential HREs in the UGP2 locus (located at positions -72 and +793, within the promoter region and first intron respectively) and one in the GBE1 locus (located within the first intron 8746 bp downstream of the transcription start site). UGP2 encodes for UTP:glucose-1-phosphate uridylyltransferase, an enzyme that synthesizes UDP-glucose, the substrate used by GS for glycogen synthesis. GBE1 encodes for 1,4-α glucan branching enzyme, the activity responsible for the generation of the highly ramified structure of glycogen. To investigate whether these genes were induced by hypoxia, we exposed c2c12 myoblasts and primary hepatocytes to 1% oxygen and determined the level of their mRNA by quantitative PCR. As shown in figure 8A, GBE1 mRNA, but not UGP2 mRNA, was strongly induced in c2c12 cells. In agreement with our data, a recent work demonstrated that GBE1 is induced by nickel treatment in a HIF-dependent manner [29]. On the other hand, UGP2 mRNA was upregulated by hypoxia in hepatocytes while GBE1 remained unaffected (figure 8A). The dissimilar behaviour of these genes in each cell type is probably a consequence of metabolic differences between muscle and liver. These differences notwithstanding, our results demonstrate that hypoxia induces the expression of all the enzymes required for glycogen synthesis. Finally, we also investigated whether, in addition to increased synthesis, a reduction of glycogen degradation could contribute to the accumulation of glycogen observed during hypoxia. In agreement with this possibility, we found a modest reduction of glycogen phosphorylase (GP) activity in cells exposed to hypoxia (figure 8B). In addition, this effect was observed even when the activity was assayed in the presence of the allosteric modulators caffeine and AMP that inhibit and activate GP, respectively (figure 8B). The effect of hypoxia on GP activity was reproducible and similar in magnitude to that induced by caffeine and AMP, but differences did not reach statistical significance. Importantly, and in sharp contrast with the GS activity data, the reduction in GP activity did not correlate with altered gene expression, since none of the PYG isoforms encoding for glycogen phosphorylase were affected by hypoxia (figure 8C). Altogether, these results indicate that hypoxia leads to glycogen accumulation by affecting different reactions involved in its metabolism.

### Hypoxia-induced glycogen accumulation promotes cell survival

**Figure 9:**
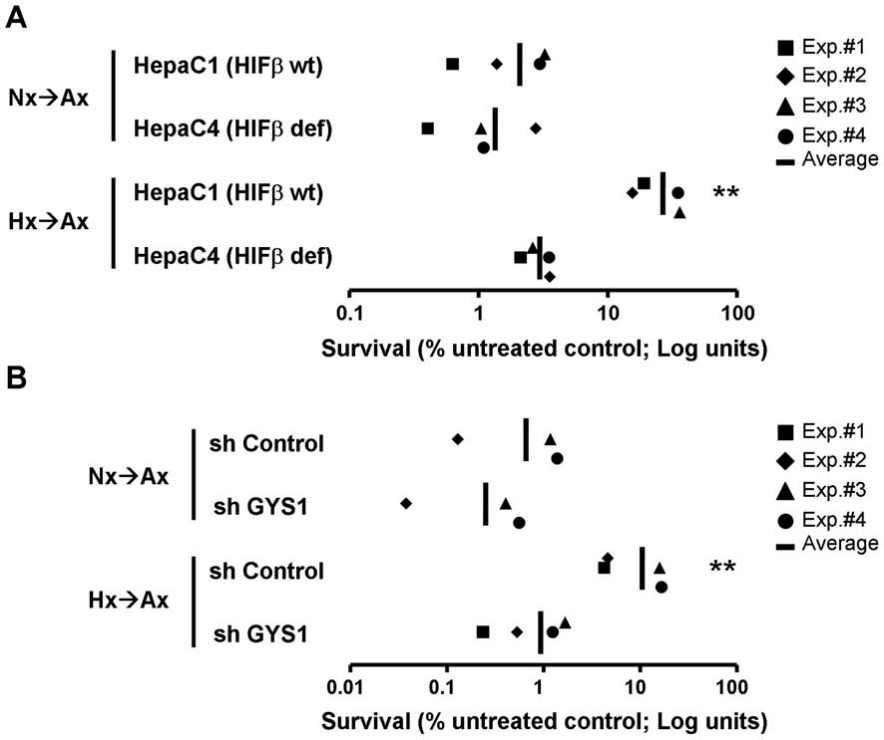
GYS1 contributes to hypoxic preconditioning. Hepa C1 and Hepa C4 cells (A) or Hepa C1 cells stably expressing a shRNA directed to GYS1 or a control shRNA (B) were exposed to 21% (Nx->Ax) or 1% (Hx->Ax) for 24 hours in complete media and then cultured in a glucose-free serum-free media and anoxia for 12 additional hours. Cell viability was determined as indicated in Materials and Methods. The results from four independent experiments and their mean are shown.

Cellular responses to hypoxia promote adaptation to low oxygen so that cells and tissues are better equipped to confront future oxygen restrictions. Therefore, we speculated that glycogen accumulation could contribute to the cellular adaptation to hypoxia by ensuring glucose supply for anaerobic glycolysis. To test this possibility, we first investigated the effect of hypoxia pre-treatment on the survival of cells deprived of glucose and exposed to severe hypoxia. As shown in figure 9A, HepaC1 cells die within 12 hours after exposure to anoxia in the absence of glucose (Nx→Ax). Importantly, exposure of cells to hypoxia (1% oxygen for 24 h), prior to the anoxic treatment in the absence of glucose (Hx→Ax), resulted in increased survival (figure 9A). Thus, in this experimental setting, hypoxia triggers a response that improves cell survival upon subsequent hypoxic challenges (hypoxic preconditioning). Moreover, as observed in other systems, hypoxic preconditioning was dependent on HIF activity since it was not observed in HIF deficient HepaC4 cells (figure 9A). Next, we asked whether glycogen accumulation could be part of the HIF-dependent responses that mediate this preconditioning. To answer this question, we prevented hypoxia-induced glycogen accumulation in HepaC1 cells by expression of a short-hairpin RNA against GYS1, shGYS1 (figure 7D and E) and determined cell viability after exposure to anoxia in the absence of glucose. In agreement with a role for GYS1 and glycogen accumulation in hypoxic preconditioning, GYS1 repression resulted in decreased viability (figure 9B). Thus, these results suggest that glycogen accumulation plays a role in the adaptive response to hypoxia.

## Discussion

The data presented herein demonstrates that GYS1 is a novel hypoxia-inducible gene. The induction of GYS1 by hypoxia requires HIF-activity and an HRE motif located on GYS1 promoter. Consequently, GYS1 should be included among the growing cohort of HIF-target genes involved in glucose metabolism. In this regard, it is interesting to note that, similar to other HIF-regulated glycolytic genes [30], GYS1 promoter is induced by the HIF1α, but not the HIF2α isoform (Figure S2).

Importantly, the induction of GYS1 by HIF results in increased total GS activity during hypoxia without altering the I/T ratio. Thus, our results indicate that the increased GS activity is a consequence of the incremented amount of enzyme, rather than of a shift in its activation state. This result is significant since it underlines the importance of GS transcription in the regulation of glycogen synthesis, as opposed to the well-characterized posttranslational modifications of GS in response to hormones and metabolites. In addition, the effect of hypoxia is highly specific, as it affects GYS1, but not GYS2, gene transcription. To our knowledge, this is the first report of transcriptional regulation of GYS1 in response to environmental cues and the first report demonstrating the regulation of glycogen synthesis at the gene expression level. Moreover, recent findings demonstrate that the blockage of GYS1 degradation, caused by laforin or malin mutations, leads to increased glycogen levels in myoclonus epilepsy [31]. Hence, alteration of GS level by modifications of its transcription and/or degradation rate constitutes a novel form of regulation of the glycogen metabolism.

Importantly, we found that, in addition to GYS1, hypoxia also induced the expression of UGP2 and GBE1, genes that encode for the rest of the enzymatic activities required for glycogen synthesis. The regulation of GBE1 was previously described [29], but, to our knowledge, this is the first report suggesting UGP2 regulation by hypoxia. In agreement with our results, analysis of public gene expression datasets (Gene Expression Omnibus at NCBI, http://www.ncbi.nlm.nih.gov) reveals upregulation of UGP2 and GBE1 in several cell types exposed to hypoxia (Ortiz et al. Nucl. Acids Res. *in press*). Further work is required to address the role of HIF in their induction by hypoxia and to determine the functionality of the identified HREs. In spite of the central role of GS as the rate limiting step in glycogen accumulation, the induction of these other genes surely contributes to an increased flow through this biosynthetic pathway. Additional experiments would be needed to address the relative contribution of these enzymes to hypoxic glycogen accumulation. On the other hand, our data suggest that hypoxia does not only regulate glycogen synthesis but also represses its degradation. This finding is in agreement with previous observations suggesting dowregulation of GP activity during prolonged hypoxia [23]. Interestingly, the reduction in GP activity was not accompanied by decreased expression of the genes coding for the enzymes bearing this activity. However, GP activity from hypoxic cells was reduced even in the presence of the allosteric activator AMP, suggesting lower protein levels. In agreement with this possibility, preliminary experiments show a reduction in protein levels of the muscle isoform of GP in hypoxic c2c12 cells (data not shown). Thus it is plausible that hypoxia affects GP translation rate and/or protein stability.

Collectively, these results indicate that hypoxia induces a coordinated response that affects glycogen metabolism at multiple levels, in a similar fashion to the paradigmatic hypoxic regulation of glycolisis. Finally, we cannot rule out that other hypoxia-triggered pathways play a role in the accumulation of glycogen. In this regard, it is likely that the upregulation of the glucose transporter SLC2A1 (Glut-1) by HIF contributes to the accumulation of glycogen by increasing the intracellular concentration of glucose.

Since anaerobic glycolysis is characterized by increased glucose demand, the induction of glycogen synthesis during hypoxia might seem paradoxical. However, as the induction of glycogen synthesis requires HIF-dependent gene expression, it will be effective during chronic hypoxia and/or subsequent hypoxic episodes. Therefore, the induction of glycogen synthesis during prolonged hypoxia might serve to replenish glycogen stores that could have been mobilized during early acute hypoxia. In agreement, it was previously proposed [22], [23] that muscle glycogen supercompensation, the accumulation of glycogen above basal levels observed after depletion of reserves during intense muscle exercise, was a consequence of the hypoxia-induced glycogen accumulation. Additionally, the induction of glycogen synthesis during chronic hypoxia results in larger cellular reserves. Our cell viability results suggest that hypoxic glycogen accumulation moderately improves cell survival following a hypoxic/isquemic insult. It should be noted however, that our assay could reflect a higher metabolic rate rather than enhanced viability. At any rate, this result implies that hypoxia-exposed cells are better equipped to respond to subsequent hypoxic challenges due to, at least in part, their higher glycogen content. In accord, hypoxia tolerant animals present large glycogen stores that are critical to ensure glucose supply during oxygen restriction [1]. Thus, we propose that the induction of GYS1 by HIF constitutes a cellular response involved in long-term adaptation to hypoxia that prepare cells to better cope with future oxygen restrictions.

Understanding the molecular mechanism of this novel response could be beneficial, not only in elucidating the physiology of the adaptations to hypoxia, but also in rationally designing novel therapeutic strategies to treat pathologies that course with hypoxia. In this regard, excessive accumulation of glycogen causes several pathologies that affect heart and central nervous system physiology. Hence, it is possible that GYS1 induction, while being an adaptive response that protects cells, could lead to an excessive glycogen accumulation in heart or brain that could contribute to damage in these organs following ischemic insults. In agreement with this possibility, it has been recently reported that chronic intermittent hypoxia leads to liver injury due, at least in part, to glycogen accumulation in hepatocytes [32]. On the other hand, our results showing that hypoxia-induced glycogen accumulation protects cells from subsequent ischemia could be relevant to tumor biology. As a consequence of aberrant vasculature, tumors are exposed to intermittent hypoxia that could induce glycogen accumulation. If this response is present, it could contribute to the resistance to fluctuations in blood supply that is commonly observed in tumors. Further work is required to test this hypothesis and to investigate the potential role of GYS1 as a target for therapeutic intervention in cancer.

In summary, the results presented herein reveal a novel metabolic adaptation of cells to function under reduced oxygen tensions. Our results further emphasize the central role of the metabolic adaptation in the response to hypoxia and the anticipatory nature of this response, as it seeks to prevent or reduce the effects of a more severe hypoxia.

## Materials and Methods

*Cell Culture and Reagents—* c2c12 myoblasts were grown in Dulbecco's modified Eagle's medium (DMEM) supplemented with 10% fetal bovine serum. To induce myogenic differentiation (c2c12 myotubes), cells were allowed to reach confluence and were then cultured in DMEM supplemented with 2% horse serum. Neonatal hepatocytes were kindly provided by Angela Martínez Valverde (IIB, Madrid). HepaC1 and HepaC4 cells were maintained in α-modifed Eagle's medium (Biochrom) supplemented with 10% fetal bovine serum. In all cases, culture medium was supplemented with 100 units/ml penicillin and 100 µg/ml streptomycin. Cells were grown at 37°C and 5% CO2 in a humidified incubator. For hypoxia treatments, cells were grown at 37°C in sealed chambers (Billups-Rothenberg) flushed with a 1% O2, 5% CO2, 94% N2 gas mixture or in a Whitley hypoxystation (don Whitley Scientific, UK) set at the indicated oxygen concentration. Dimethyloxalylglycine (DMOG, Frontier Scientific, CA,USA) was added to the indicated cultures at a 250 µM final concentration.

*Plasmid Construction-* Human genomic DNA extracted from human cervical carcinoma (HeLa) cells was used as template for PCR amplification of GYS1 promoter regions. PCR products were first cloned into pCR2.1-TOPO (invitrogen) and subsequently subcloned for reporter assays into the KpnI/XhoI restriction sites of pGL3-Basic (Invitrogen) or the BamHI site of prolactin- plasmid [33], in both possible orientations. The identity of all constructs was verified by sequencing. Primer sequences are available from the authors upon request.

*Reporter assays-* Reporter assays were performed using the human cervical-carcinoma cell line HeLa. Cells were seeded on six-well plates (3.5*104 cells/well) 6 h prior to transfection. A 9* µ*g DNA mixture containing 3* µ*g of the indicated reporter construct or empty plasmid and 0.1* µ*g of a plasmid encoding for *Renilla* (sea pansy) luciferase under the control of a null promoter (Promega, Madison, WI, U.S.A.) was used for transfection using the calcium phosphate method. 16 h after transfection, cells where washed, replated in 24-well plates, and incubated in normoxia, in the presence of DMOG or in hypoxia for an additional 16 hours. After treatments cells were lysed and the firefly and *Renilla* luciferase activities of the lysate were determined using a dual-luciferase system (Promega, Madison, WI, U.S.A.). The firefly luciferase activity was normalized to that of *Renilla* luciferase.

*Treatment with Recombinant Adenoviruses*— AdCMV-GFP-MGS and AdCMV-galactosidase were described previously [34]. For infections cells were incubated for 2 h with an appropriate amount of adenovirus in OptiMEM. Infection medium was then replaced with DMEM, and cells were incubated for 48 h at 37°C in a humidified incubator.

*RNA interference*- We used the following commercial siRNAs: HIF1alpha siRNA (Santa Cruz, predesigned siRNA#16708), GYS1 siRNA (Qiagen, SI01060843), and All Stars siRNA (Qiagen, catalog # 1027281) as a negative control. A concentration of 30 nM of each siRNA was transfected into c2c12 cells using Lipofectamine 2000 (Invitrogen), following the manufacturer's instructions. The efficiency of HIF1alpha or GYS1 knockdown was determined by real time quantitative PCR analysis. Stable attenuation of GYS1 expression was achieved by selection of pooled cells infected with lentivirus expressing a short hairpin RNA directed to mouse GYS1. The lentiviral constructs were a kind gift from Carles Martinez-Pons (IRB, Barcelona).

*Metabolite determinations*- Glycogen determination was performed as previously described [35]. Briefly, cells were scraped with 30% (w/v) KOH; the extract was then boiled for 15 min and the resulting solution was spotted on chromatography paper 31 ET (Whatman). Glycogen was precipitated by immersing the papers in ice-cold 66% (v/v) ethanol. After two washes in ethanol, the papers were air-dried and incubated with amyloglucosidase (Sigma). The resulting glucose was measured with a Gluco-quant kit (ABX entra). The amount of glycogen is represented as the amount of released glucose per mg of total protein. L-Lactate levels in the incubation medium were measured as described [36].

*GS and GP activity determination*- To measure GS and GP activities, frozen plates were scraped with 300* µ*l of homogenization buffer consisting of 10 mM Tris/HCl (pH 7.0), 150 mM KF, 15 mM EDTA, 600 mM sucrose, 15 mM 2-mercaptoethanol, 10* µ*g/ml leupeptin, 1 mM benzamidine and 1 mM PMSF, and the collected cells were subsequently sonicated. The resulting homogenates were used for the determination of enzymic activities. Protein concentration was measured by the Bio-Rad protein assay. GP activity was determined by measuring the incorporation of [U-14C]glucose 1-phosphate into glycogen in the absence or presence of AMP (5 mM) as describe previously [37]. The GS activity ratio was determined by measuring the incorporation of [U-14C]UDP-glucose into glycogen in the absence or presence of 6.6 mM glucose 6-phosphate, as described previously [38]. The activity measured in the absence of Glc-6-P represents the active form of the enzyme (I or *a* form), whereas that measured in the presence of 6.6 mM Glc-6-P represents total GS activity.

*Western Blot—*Immediately after treatments, cells were washed with ice-cold phosphate-buffered saline and harvested in 70–200 µl of 1x Laemmli sample buffer (2%SDS, 10% glycerol, 5% 2-mercaptoethanol, 0.002% bromphenol blue and 0.125 M Tris HCl pH 6.8). Lysates were sonicated for 4 s, centrifuged at 4°C for 2 min at 14,000×*g*, and resolved on 8–10% SDS-polyacrylamide gels. Proteins were then transferred to nitrocellulose membranes (Bio-Rad), blocked with 5% nonfat dry milk in TBS-T (50 mM Tris, pH 7.6, 150 mM NaCl, 0.1% Tween 20), and incubated overnight at 4°C with the indicated antibodies (HIF1α was from R&D systems, ref. MAB1536 and GYS1 was from Cell Signalling, ref 3893). Immunoreactive bands were visualized with the Amersham Biosciences ECL system.

*ChIP (chromatin immunoprecipitation)*- For ChIP assays, c2c12 cells were grown on 10 cm plates until they reached 85% confluence, at which point they were exposed to hypoxia (1% oxygen) or left under normoxic conditions for a further 5 h. Subsequently, cells were fixed with 1% (v/v) formaldehyde (final concentration) for 12 min at 37°C. Crosslinking was stopped by the addition of 0.125 M glycine (final concentration).The cells were washed with cold PBS and then lysed by scraping in 1 ml of lysis buffer (1% SDS, 10 mM EDTA, 50 mM Tris/HCl, pH 8.1, and a protease inhibitor cocktail, Roche). Cell lysates were incubated on ice for 10 min and then sonicated to shear the DNA under conditions established to ensure that the DNA fragments were between 200 and 1500 bp. After the removal of the insoluble material by centrifugation, 30* µ*l of each sample was removed and stored (input), while the rest was diluted in immunoprecipitation buffer (1% Triton X-100, 2 mM EDTA, 150 mM NaCl and 20 mM Tris/HCl, pH 8.1). The lysates were precleared with preimmune serum and 200* µ*g of a Salmon Sperm DNA/Protein A agarose 50% slurry (Upstate Biotechnology, Lake Placid, NY, U.S.A.) for 1 h at 4°C. The samples were then immunoprecipitated twice, initially with whole rabbit serum for 6 h (IgG control) and then overnight at 4°C with a polyclonal anti-HIF1alpha antiserum (Abcam, ab2185). Immunocomplexes were recovered by the addition of 400* µ*g of Salmon Sperm DNA/Protein A agarose 50% slurry to the samples that were then sequentially washed for 15 min in TSE I (0.1%SDS, 1%Triton X-100, 2 mM EDTA, 20 mM Tris/HCl, pH 8.1, and 150 mM NaCl), TSE II (0.1% SDS, 1% Triton X-100, 2 mM EDTA, 20 mM Tris/HCl, pH 8.1, and 500 mM NaCl) and buffer III (0.25 M LiCl, 1% Nonidet P40, 1% deoxycholate, 1 mM EDTA and 10 mM Tris/HCl, pH 8.1). Finally, the complexes were washed twice with TE buffer (10 mM Tris, pH 8.0, and 1 mM EDTA) and extracted twice with a buffer containing 1% SDS and 0.1 M NaHCO3. The eluates were pooled, and cross-linking was reversed by the addition of 200 mM NaCl (final concentration) and overnight incubation at 65°C. The proteins were removed by the addition of proteinase K (30* µ*g/sample) for 2 h at 42°C, and the DNA was extracted using Qiagen PCR extraction kit before eluting in 50* µ*l of water. Immunoprecipitated DNA was amplified by PCR using the primers indicated:

GYS1F:TCGGATCCGGTACCGCTTTACGGAAACGAGTG

GYS1R:TACTCGAGGGTACCGAACTCCTGGGTCCATTC

P4HaF: ATCAAGGAGGCAAACTGAACAG

P4HR: ACTCGGAGCGGCTACTTCCTA

The PCR products were resolved by gel electrophoresis and visualized by ethidium bromide staining.

*RNA Extraction and Quantitative PCR—* Total RNA was extracted and purified with the RNeasy Mini Kit (Qiagen). 1 µg of total RNA from each sample was retrotranscribed to cDNA (Improm-II reverse transcriptase; Promega). 1–3 µl of cDNA samples were used as template for amplification reactions carried out with the LC Fast Start DNA master SYBR Green I kit (Roche Applied Science) following the manufacturer's instructions. PCR amplifications were carried out in a Light Cycler System (Roche Applied Science), and data were analyzed with LightCycler software 3 version 3.5.28 (Idaho Technology Inc.). For each sample, duplicate determinations were made, and the gene expression determined by the ΔΔCt method using β-actin as reference gene. The primer used in this study are (5′-3′): GYS1, forward (TTCTACAACAACCTGGAG) and reverse (CTGAGCAGATAGTTGAGC); GYS2, forward (TTACCAGCATGCCAGACAC) and reverse (AGAAGGTGGTACTGAGG); HIF 1alpha , forward (GTTTACTAAAGGACAAGTCACC) and reverse (TTCTGTTTGTTGAAGGGAG); β-actin forward (CCCAGAGCAAGAGAGG) and reverse (GTCCAGACGCAGGATG). UGP2, forward (CAGAGACCTCCAGAAGATTCG) and reverse (GTTCAACACAGAAGATATGTTATCAGG); GBE1, forward (GCTCGGTGGAGAAGGCTAT) and reverse (CTTGGGAAGTCCAACCATTC).

*Cell viability assay*.- HepaC1, HepaC4, HepaC1-Ad1 (stably expressing a control shRNA) and HepaC1-Ad5 cells (stably expressing a shRNA directed to mouse GYS1) were seeded in 96-well plates at a density of 5×10^3^/well in 100 µl of complete medium. Then the cells were incubated in normoxia or in hypoxia for 24 h. After treatments culture media was removed, cells were washed, changed to glucose and serum free medium, and incubated in normoxia or in anoxia for additional 16 hours. Cell viability was determined with the CellTiter-Glo cell viability assay (Promega, WI). Luciferase activity was detected in a microplate luminometer reader (E6521;Promega,WI).

*Statistical analysis of data*– Experimental data were analyzed with the Prism™ GraphPad (version 4.01) software. Data were analyzed by the analysis of variance test (ANOVA) followed by the Tukey's or Dunnett's multiple comparison test. Significant differences with control values (indicated in each figure legend) are shown by asterisks.

## Supporting Information

Figure S1 GYS1 induction in different cell types. The indicated cell lines were exposed to normoxia or hypoxia for 6 hours and the level of GYS1 mRNA was determined by quantitative PCR. The amount of each mRNA in samples was normalized to the content of beta-actin mRNA in the same sample. Data shown represent the fold values of hypoxic over normoxic mRNA levels normalized to the value of 1 (horizontal line). The experiment was repeated twice with similar results.
(1.24 MB TIF)

Figure S2 Promoter activation by HIF1a or HIF2a overexpression. HeLa cells transfected with a reporter plasmid containing BNIP3 (A), CITED2 (B), VEGFA (C) or GYS1 (D) promoters upstream a luciferase reporter gene alone (none) or in combination with constructs encoding for HIF1a or HIF2a. The graphs represent the corrected luciferase activity values of each construct over the luciferase activity obtained in normoxic cells transfected with empty plasmids. Data shown are the average results of three independent experiments and error bars the standard deviation.
(2.33 MB TIF)
