# Supplementary figures and images for: Hypoxia Promotes Glycogen Accumulation through Hypoxia Inducible Factor (HIF)-Mediated Induction of Glycogen Synthase 1
Source: PLoS One. 2010 Mar 12;5(3):e9644. doi: 10.1371/journal.pone.0009644 (PMC2837373; doi:10.1371/journal.pone.0009644)

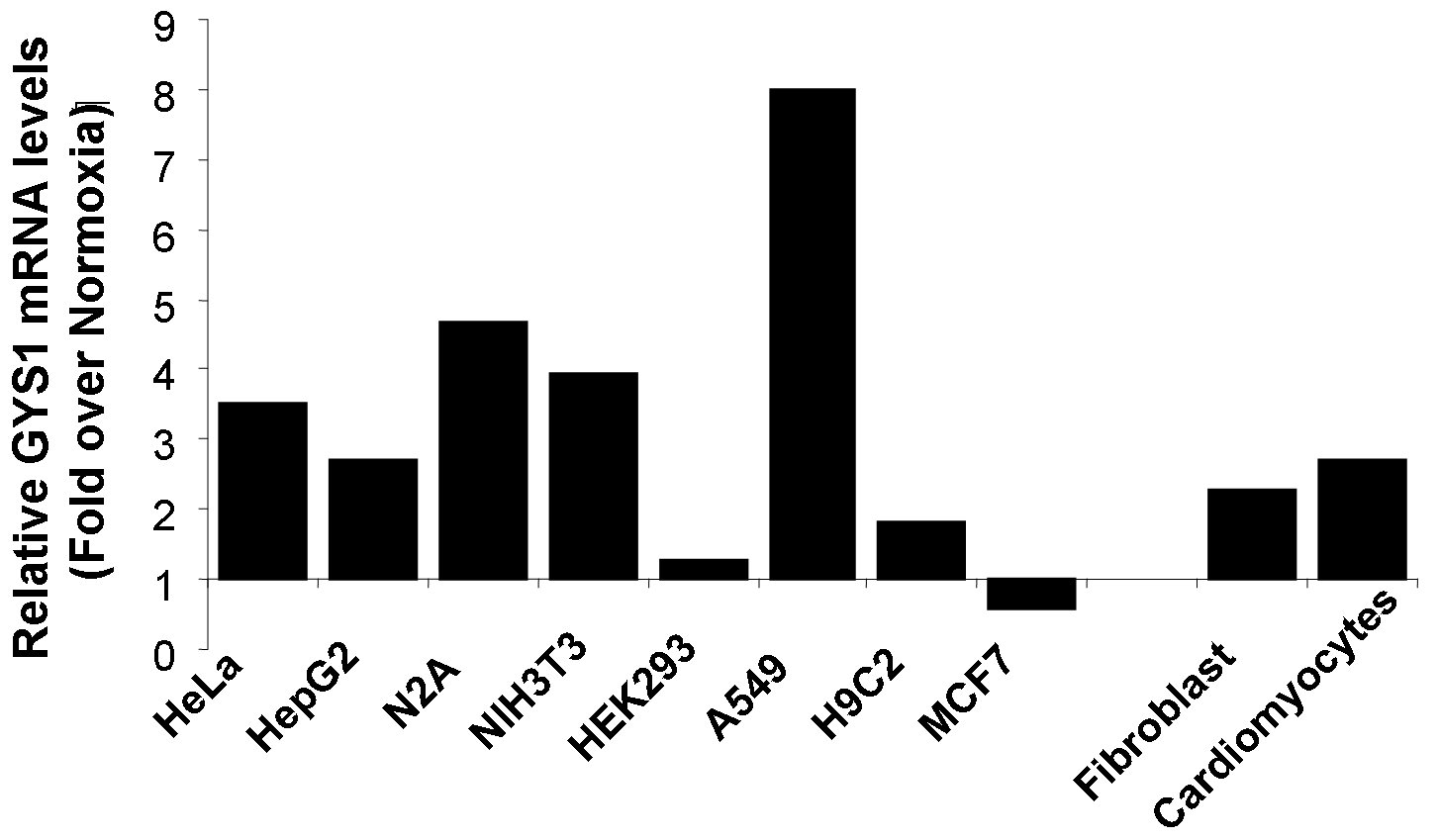

Supplement: Figure S1 — GYS1 induction in different cell types. The indicated cell lines were exposed to normoxia or hypoxia for 6 hours and the level of GYS1 mRNA was determined by quantitative PCR. The amount of each mRNA in samples was normalized to the content of beta-actin mRNA in the same sample. Data shown represent the fold values of hypoxic over normoxic mRNA levels normalized to the value of 1 (horizontal line). The experiment was repeated twice with similar results. (1.24 MB TIF) [file pone.0009644.s001.tif]

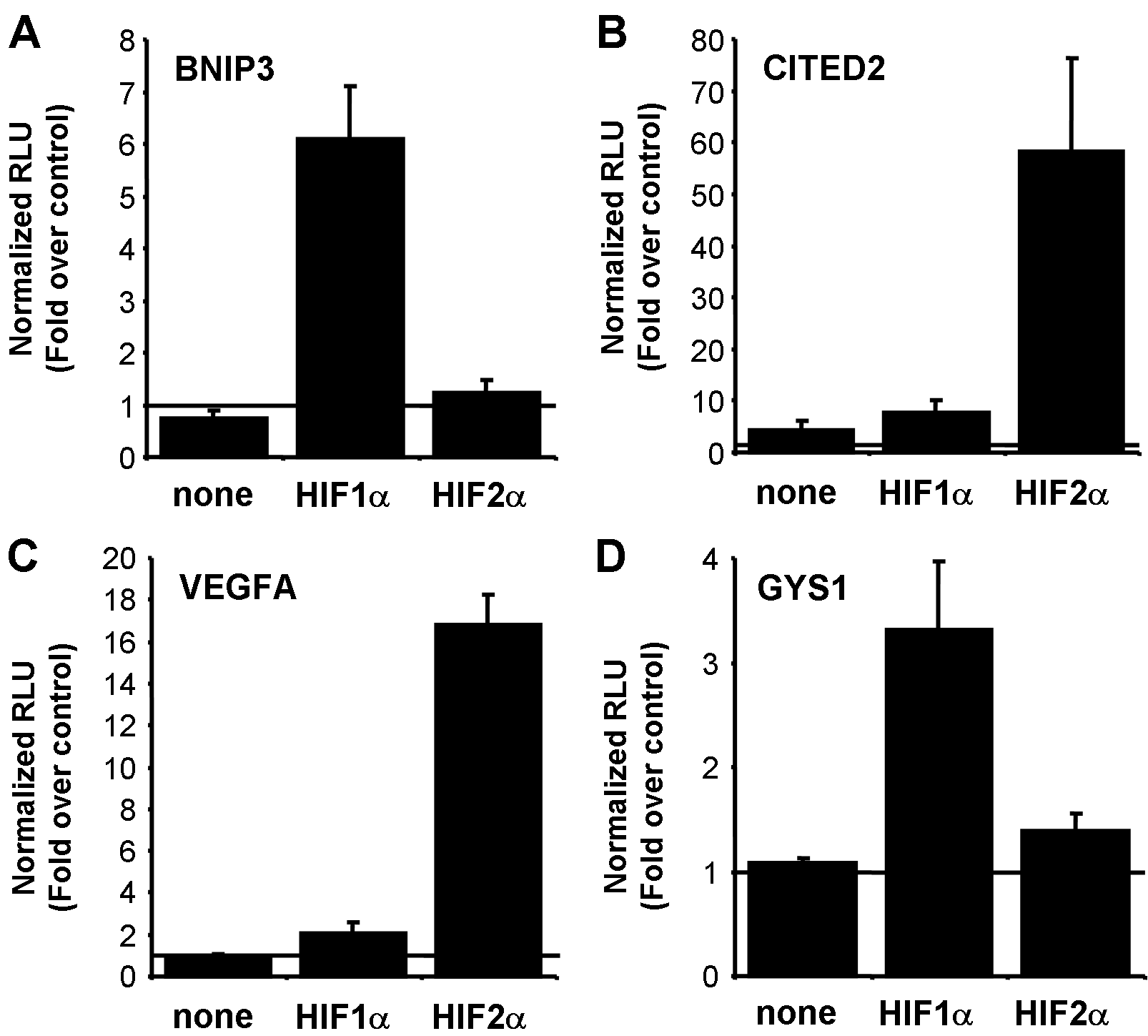

Supplement: Figure S2 — Promoter activation by HIF1a or HIF2a overexpression. HeLa cells transfected with a reporter plasmid containing BNIP3 (A), CITED2 (B), VEGFA (C) or GYS1 (D) promoters upstream a luciferase reporter gene alone (none) or in combination with constructs encoding for HIF1a or HIF2a. The graphs represent the corrected luciferase activity values of each construct over the luciferase activity obtained in normoxic cells transfected with empty plasmids. Data shown are the average results of three independent experiments and error bars the standard deviation. (2.33 MB TIF) [file pone.0009644.s002.tif]
